# Gut microbiota metabolites and key target molecules in allergic rhinitis: a multi-omics study of gut-nose axis regulation via the inflammation-metabolism network

**DOI:** 10.3389/fmicb.2025.1702518

**Published:** 2025-11-27

**Authors:** Jingqi Zhang, Mengqi Zhao, Ya Yu, Yining Zeng, Meiyi Sun, Chuanyu Wu, Tao Guo, Liping Lin, Zhiqing Liu, Hui Xie

**Affiliations:** 1 Department of Otolaryngology-Head and Neck Surgery, Hospital of Chengdu University of Traditional Chinese Medicine, Chengdu/Sichuan, China; 2 Chengdu University of Traditional Chinese Medicine, Chengdu/Sichuan, China

**Keywords:** gut microbiota-derived metabolites, allergic rhinitis, core genes, target prediction, gut-nasal metabolic axis

## Abstract

**Introduction:**

Allergic rhinitis (AR) is a globally prevalent immune-mediated disorder. While the “gut-nasal axis” suggests that gut microbiota metabolites can modulate AR, the specific molecular networks and key targets involved remain poorly defined. This study aimed to systematically explore the molecular connections between gut microbiota-derived metabolites and AR, and to identify potential therapeutic targets.

**Methods:**

We performed an integrative multi-omics analysis using databases including gutMGene and GeneCards to identify overlapping genes. Summary-data-based Mendelian randomization (SMR) was used to investigate associations between genetic variation, DNA methylation, gene expression, and AR. Single-cell analysis was conducted to examine gene expression patterns in the AR nasal mucosa. Finally, upstream analysis, target prediction, and molecular docking were employed to identify key metabolites, protein targets, and candidate drugs.

**Results:**

We identified 20 overlapping genes, highlighting a significant association between AR and core inflammatory mediators like IL6, TNF, IL1B, and IL4R. SMR analysis indicated that genetic and epigenetic regulation within the interleukin gene family is closely linked to AR. Single-cell analysis revealed distinct expression patterns of these core genes in the nasal mucosa. Upstream analysis connected these findings to specific microbiota-derived metabolites, notably indole-3-propionic acid and succinate. MPO and PTGDR2 were identified as key potential targets, and Fevipiprant and Zileuton were proposed as candidate drugs.

**Conclusion:**

This study provides the first systematic exploration of the “gut-nasal” metabolic axis in AR at a multi-level molecular network level, offering novel perspectives on the disease's underlying mechanisms. The identified targets and candidate drugs provide a valuable foundation for developing new therapeutic strategies, warranting further experimental validation for potential clinical translation.

## 1 Introduction

Allergic rhinitis (AR) is a chronic inflammatory disease of the nasal mucosa driven by type 2 helper T cell (Th2) immune responses. It affects approximately 15% of the United States population, with a steadily rising prevalence, and has become a significant public health concern (Bernstein et al., 2024). Hallmark pathological features include eosinophilic infiltration, IgE-mediated activation of mast cells, and excessive secretion of proinflammatory cytokines such as IL-4, IL-5, and IL-13, which manifest clinically as nasal pruritus, rhinorrhea, and congestion (Wise et al., 2018; Siddiqui et al., 2022). Current therapies (antihistamines, intranasal corticosteroids, and allergen immunotherapy) primarily alleviate symptoms without providing a cure, and long-term use can lead to adverse effects, including epistaxis and steroid dependence (Seidman et al., 2015). Thus, there is an urgent need to uncover new pathogenic mechanisms and identify effective, low-toxicity therapeutic targets.

In recent years, the immunoregulatory roles of the gut microbiota and its metabolites have garnered substantial attention. Evidence indicates that the gut microbiota produces small molecules such as short-chain fatty acids (SCFAs), tryptophan metabolites (e.g., indole derivatives), and bile acids that directly or indirectly modulate the host immune system (Levy et al., 2017; Rooks and Garrett, 2016). For example, butyrate promotes regulatory T cell (Treg) differentiation via activation of G protein-coupled receptor 41 (GPR41) and G protein-coupled receptor 43 (GPR43), thereby suppressing Th2-skewed immunity (Furusawa et al., 2013; Smith et al., 2013). Indole-3-propionic acid (IPA), an aryl hydrocarbon receptor (AhR) ligand, can inhibit Nuclear Factor kappa B (NF-κ B) signaling and may mitigate airway inflammation (Wlodarska et al., 2017; Venkatesh et al., 2014). Notably, microbiota-derived metabolites may regulate mucosal immunity at distal sites through the “gut-lung” or “gut-nasal axes”, offering a new perspective on the long-range regulation of AR (Budden et al., 2017). The “gut-lung axis” is founded on the structural homology between the lungs and intestines, which share a common embryonic origin from the endoderm (Tichelaar et al., 1999). This developmental link is so profound that altering transcription factor activity can induce intestinal progenitors from embryonic lung progenitors (Okubo and Hogan, 2004). Functionally, this axis involves bidirectional communication mediated by gut microbial metabolites circulating in the bloodstream. Notably, short-chain fatty acids (SCFAs) play a key role in mucosal immune responses by enhancing plasmablast metabolism (Wu et al., 2017). For example, mice on a high-fiber diet have higher circulating SCFA levels and demonstrate protection against allergic lung inflammation (Byndloss et al., 2017). Dysregulation of this axis is implicated in allergic airway diseases. Delayed maturation of the gut microbiota in early life is a known risk factor for allergic rhinitis (AR) (Hoskinson et al., 2023), and associated disturbances in metabolite profiles contribute to immune dysregulation along the “gut-nose axis” (Hong et al., 2025). While the exact mechanisms are still being elucidated, studies offer key insights. For instance, PM2.5 particles can trigger gut microbial dysbiosis, leading to NLRP3 inflammasome activation and the subsequent exacerbation of nasal epithelial barrier damage in AR (Li et al., 2025). While such studies highlight associations between microbial composition and disease, the specific molecular mechanisms—namely, how microbial metabolites regulate AR through host gene networks—remain poorly understood.

Multiple signaling pathways underlying AR have been identified, including innate immune activation via the Toll-Like Receptor (TLR)/Myeloid Differentiation primary response 88 (MyD88)/NF-κB axis and Th2 polarization mediated by Janus kinase-signal transducer and activator of transcription (JAK-STAT) signaling (Galli et al., 2008). Their interactions with gut microbiota-derived metabolites, however, are not well defined. The advent of network pharmacology and bioinformatics provides powerful tools to dissect multi-target regulatory networks in complex diseases. By integrating gene-gene interactions, metabolite-target prediction, and functional enrichment analyses, these approaches can map cascade relationships among metabolites, genes, pathways, and diseases (Huang et al., 2014). Nevertheless, applications of these strategies to AR in the context of the gut microbiota are still at an early stage.

**Figure 1:**
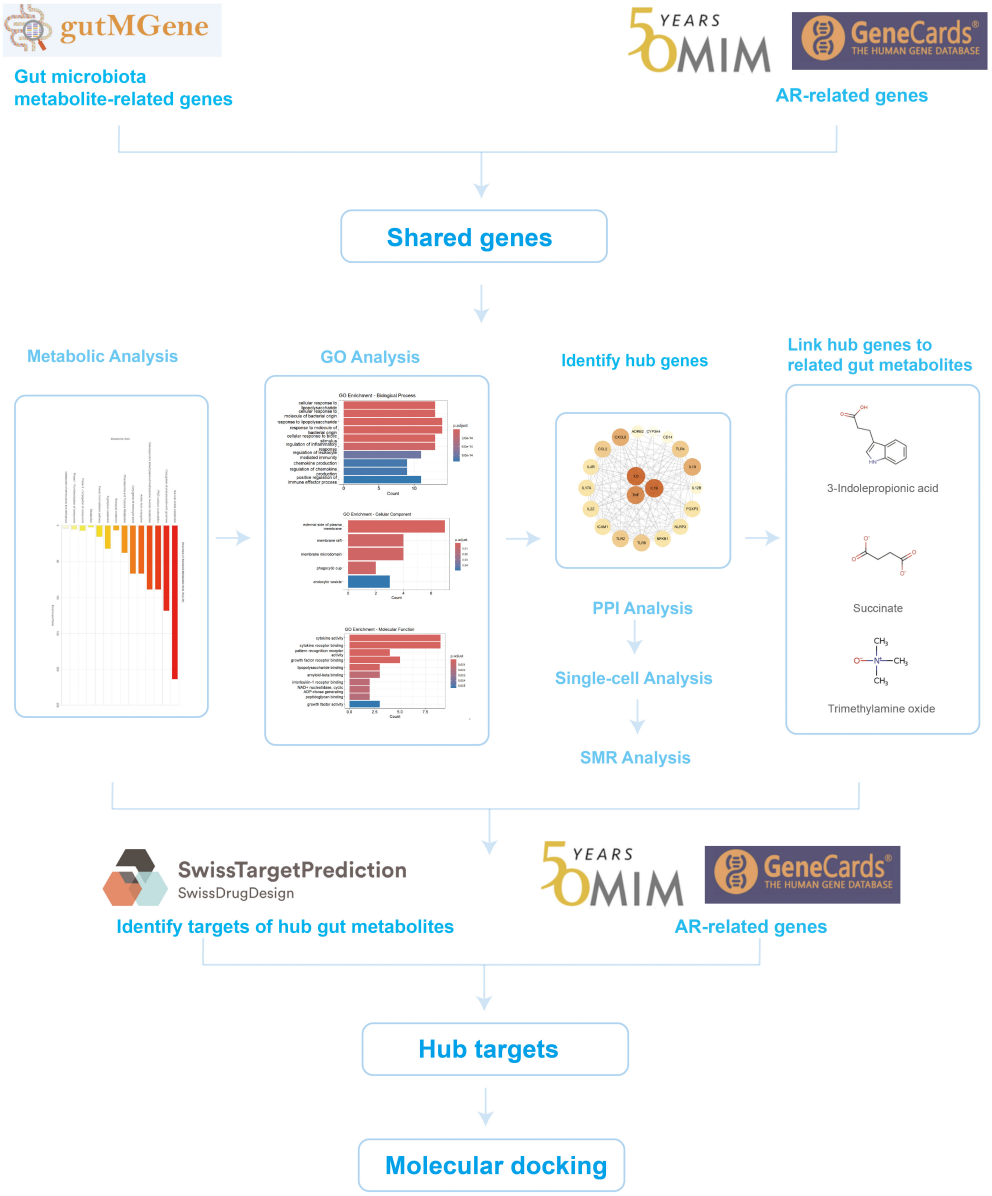
Research flow diagram.

To test this hypothesis, we developed a systematic, multilayer analytical framework integrating multi-omics data with bioinformatics approaches to elucidate how gut microbiota metabolites modulate AR. This framework first identified and prioritized key gene targets at the nexus of gut metabolite action and AR pathophysiology. We then employed a convergence of evidence approach—integrating summary-data-based Mendelian randomization (SMR) and single-cell RNA sequencing (scRNA-seq)—to establish causal relevance and characterize the cellular origins of these genes within the nasal mucosa. Building on these prioritized targets, we identified specific microbiota-derived metabolites capable of their modulation and, finally, explored the translational potential of this axis by predicting candidate drug molecules and evaluating their pharmacological properties through molecular docking and ADMET profiling (Figure 1).

Through the integration of multiple databases and multidimensional analyses, this study seeks to elucidate a comprehensive regulatory cascade linking gut microbiota-derived metabolites to core genes, signaling pathways, and therapeutic targets, thereby unveiling the potential mechanisms underlying AR. Our findings are expected to advance the theoretical understanding of AR pathogenesis and facilitate the development of metabolite-based precision interventions and novel therapeutic candidates.

## 2 Methods

### 2.1 Data acquisition and gene screening

We retrieved gut microbiota metabolite-related genes from the gutMGene database (http://gutmgene.genome.cn/), which catalogs associations among 332 human gut microbes, 207 microbial metabolites, and genes (Qi et al., 2025). AR-related genes were obtained from GeneCards (https://www.genecards.org/) by searching the keyword “Allergic Rhinitis” and retaining entries with a Relevance score >10 (Lu et al., 2022), and additional AR-related genes were collected from Online Mendelian Inheritance in Man (OMIM) (https://www.omim.org/). The GeneCards and OMIM lists were merged and deduplicated, and their intersection with the gut microbiota metabolite-related gene set was taken to yield the genes shared by AR and gut microbiota metabolites.

### 2.2 Metabolic pathway enrichment analysis

To investigate the functions and potential roles of metabolites associated with the AR-related shared genes linked to the gut microbiota, metabolic pathway enrichment was performed using MetaboAnalyst 6.0 (https://www.metaboanalyst.ca/) (Pang et al., 2024). MetaboAnalyst is a comprehensive web-based platform for processing, analyzing, and interpreting targeted and untargeted metabolomics data, and it provides dedicated modules for functional analysis.

### 2.3 GO functional enrichment analysis

Gene Ontology (GO) enrichment analysis of the shared genes was conducted in R (version 4.1.0) using the clusterProfiler package (version 4.0.5). Gene symbols were converted to Entrez IDs with org.Hs.eg.db (version 3.13.0). The analysis covered the three GO domains, namely biological process (BP), molecular function (MF), and cellular component (CC), with the significance threshold set at adjusted *p* < 0.05 using the Benjamini-Hochberg correction. For visualization, bar plots of the top 10 significantly enriched terms were generated with ggplot2 (version 3.3.5). The results highlight key biological functions and potential pathogenic mechanisms of genes jointly implicated in AR and microbiota-derived metabolites, providing clues to the molecular basis of their interaction.

### 2.4 Protein-protein interaction (PPI) network and hub gene identification

Shared genes were submitted to STRING (https://string-db.org/) to construct a protein-protein interaction network. The exported tab-separated values (TSV) file was imported into Cytoscape (version 3.10.3) for visualization, and a force-directed layout was applied to optimize the network structure. Hub genes were prioritized based on degree centrality.

### 2.5 SMR analysis

Summary data based Mendelian randomization was employed to evaluate genetic relationships, potential causal effects, and molecular mechanisms underlying AR. The validity of SMR analysis rests on three core assumptions for instrumental variables (IVs): (1) the relevance assumption, where IVs are strongly associated with the exposure (the molecular phenotype); (2) the independence assumption, where IVs are not associated with confounders; and (3) the exclusion restriction assumption, where IVs affect the outcome (AR) only through the exposure. To satisfy the relevance assumption, we used SNPs that were strong cis-acting quantitative trait loci (cis-QTLs). The primary instrumental variable for each test was the SNP showing the strongest association with its corresponding molecular phenotype (gene expression, splicing, protein abundance, or DNA methylation) in the respective QTL dataset, typically requiring a *P*-value approaching or exceeding genome-wide significance (e.g., *P* < 5 × 10^−8^).

GWAS summary statistics for AR were obtained from the IEU OpenGWAS project (ukb-b-16499; 26,107 cases and 436,826 controls) and used as the outcome dataset. SMR analyses were performed with the standalone software (smr-1.3.1-win) and the online platform (https://yanglab.westlake.edu.cn/software/smr), integrating whole-blood quantitative trait locus (QTL) resources from multiple large cohorts, including the eQTLGen consortium eQTL meta-analysis (n = 31,684), splicing QTLs from the Genotype-Tissue Expression (GTEx) project (https://gtexportal.org/home/) (*n* = 755) (“The Genotype-Tissue Expression (GTEx) project, 2013”), protein QTLs from INTERVAL (*n* = 3,301) (Sun et al., 2018) and the SCALLOP consortium (*n* = 30,931) (Folkersen et al., 2020), and methylation QTLs from McRae et al. (*n* = 1,980) (McRae et al., 2018). The analysis was run comprehensively against all molecular phenotypes (gene expression, splicing, protein abundance, or DNA methylation) available within these genome-wide QTL datasets. All QTL datasets comprised genome-wide associations between single nucleotide polymorphisms (SNPs) and molecular phenotypes (gene expression, splicing, protein abundance, or DNA methylation).

The minor allele frequency threshold for IVs was set at 0.01. For each identified association, we reported the SMR effect size (b_SMR) and the corresponding *P*-value. To distinguish causality from horizontal pleiotropy, we utilized the Heterogeneity in Dependent Instruments (HEIDI) test. An association with a *p*_HEIDI <0.05 was considered to exhibit significant heterogeneity, suggesting that the observed association might be due to linkage disequilibrium (LD) between two distinct causal variants rather than a true causal effect, and such signals were excluded from causal interpretation. This integrative framework enables systematic nomination of candidate genes that may mediate the relationship between genetic variation and AR risk and assessment of their regulatory roles at the levels of gene expression, splicing, and related molecular traits.

### 2.6 Single-cell analysis

A publicly available scRNA-seq dataset of allergic rhinitis (GSE261706), comprising AR (*n* = 2) and healthy control (*n* = 2) nasal mucosal samples, was analyzed using Seurat (version 4.0). Quality control retained cells with 200–4,000 detected genes and mitochondrial gene proportions below 10%. Data were normalized with the “LogNormalize” method, and batch effects were mitigated using the Harmony algorithm. To reveal expression changes of core genes in normal vs. AR samples, differential expression analysis was performed on clustered and annotated cells, with emphasis on the expression patterns of prioritized core genes across cell types. “FeaturePlot” visualized the distribution of these genes on the Uniform Manifold Approximation and Projection (UMAP) embedding, highlighting differences between AR and healthy nasal mucosa, while “VlnPlot” quantified expression levels across cell types to delineate AR-associated alterations. These analyses provide a systematic assessment of disease-related expression dynamics and cell-type specificity of the core genes in AR.

### 2.7 Metabolite-target association analysis

Metabolites directly associated with the prioritized core genes were extracted from the gutMGene database. For each metabolite, SMILES strings were downloaded from PubChem (https://pubchem.ncbi.nlm.nih.gov/) and submitted to SwissTargetPrediction (http://www.swisstargetprediction.ch/) with the organism set to Homo sapiens, and all predicted targets were retained. The predicted targets were then intersected with the AR-related gene set compiled from GeneCards and OMIM to define core targets potentially mediating the effects of gut microbiota-derived metabolites in AR.

### 2.8 Molecular docking validation

Protein structures for key targets were retrieved in PDB format by querying UniProt (https://www.uniprot.org/) and the RCSB Protein Data Bank (https://www.rcsb.org/). Structures were prepared in PyMOL (version 2.5) by removing water molecules and co-crystallized ligands. Candidate drugs acting on these targets were predicted using the Coremine platform (https://www.coremine.com/) and cross-checked in DrugBank (https://go.drugbank.com/) to enhance reliability. Molecular docking was performed with CB-Dock2 (http://clab.labshare.cn/cb-dock2/); binding energies ≤ -5.0 kcal/mol were considered indicative of feasible binding, whereas energies ≤ −7.0 kcal/mol reflected good binding performance (Trott and Olson, 2010). The databases used are summarized in Table 1.

**Table 1:** Online databases used in this study.

**Tools/Databases**	**Website/URL**
Gutmgen	http://bio-computing.hrbmu.edu.cn/gutmgene/
GeneCards	https://www.genecards.org/
OMIM	https://www.omim.org/
STRING	https://string-db.org/
PubChem	https://pubchem.ncbi.nlm.nih.gov/
UniProt	https://www.uniprot.org/
MetaboAnalyst 6.0	https://www.metaboanalyst.ca/
SwissTargetPrediction	http://www.swisstargetprediction.ch/
OmicsNet	https://www.omicsnet.ca/
Coremine	https://www.coremine.com/
CB-Dock2	https://cadd.labshare.cn/cb-dock2/
DrugBank	https://go.drugbank.com/

### 2.9 Drug evaluation

Drug-likeness and oral developability of predicted candidates were assessed using ADMETlab 3.0 (https://admetlab3.scbdd.com). Filtering followed Lipinski's criteria: (1) molecular weight <500 g/mol; (2) lipophilicity (logP) <5; (3) number of hydrogen-bond acceptors <5; (4) number of hydrogen-bond donors <5; (5) topological polar surface area (TPSA) <140 Å^2^. Compounds meeting all five criteria were retained for further analysis, facilitating early-stage triage of high-potential candidates and improving development efficiency.

## 3 Results

### 3.1 AR and gut microbiota-derived metabolites converge on multiple metabolic pathways

**Figure 2:**
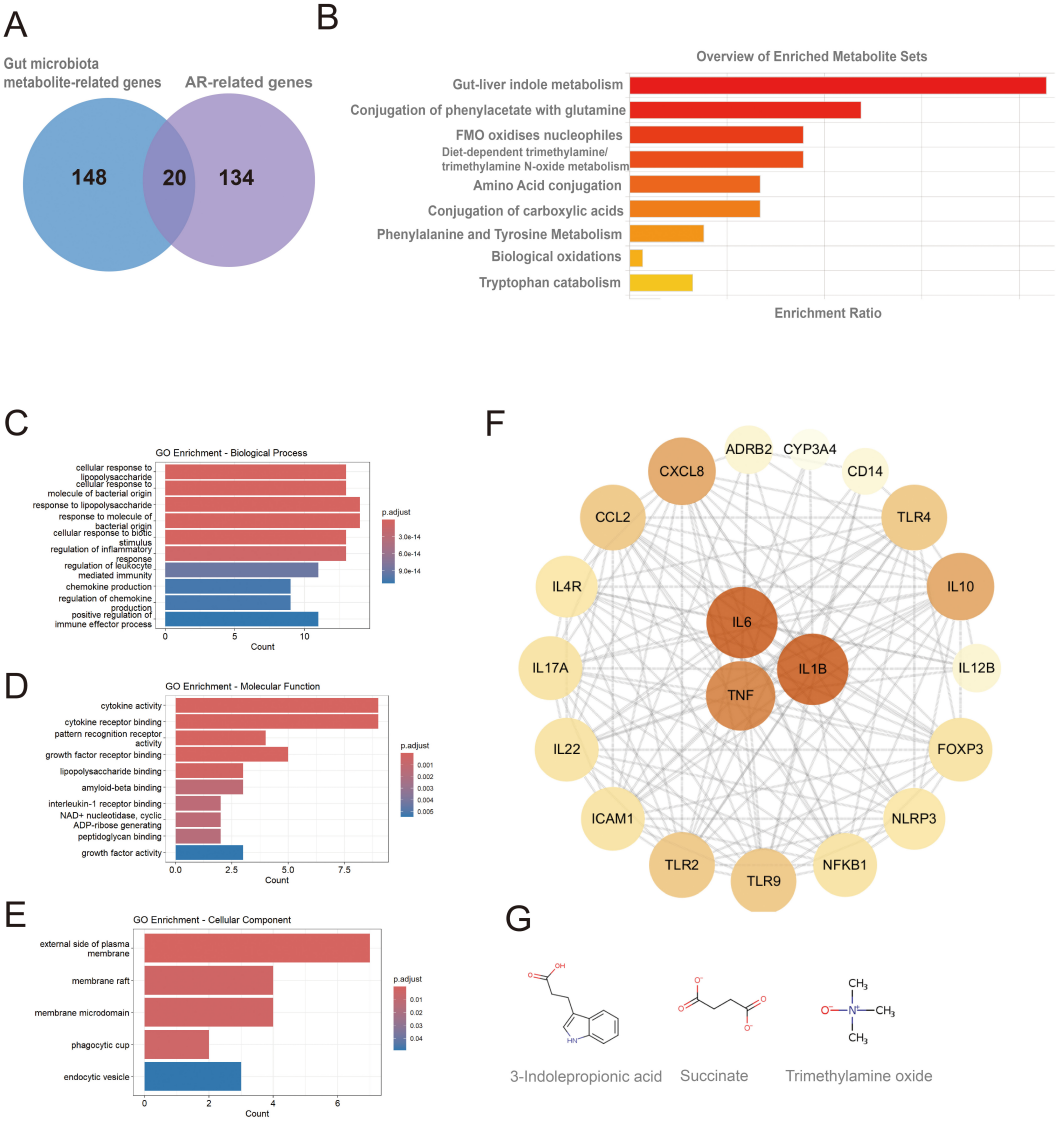
Metabolic pathway and GO enrichment analyses of shared genes between gut microbiota-derived metabolites and allergic rhinitis, and identification of hub genes. **(A)** Twenty shared genes between gut microbiota-derived metabolites and AR; **(B)** metabolic pathway enrichment of the shared genes; **(C)** GO biological process (BP) terms; **(D)** GO molecular function (MF) terms; (**E)** GO cellular component (CC) terms; **(F)** hub genes identified from the protein-protein interaction (PPI) network, node color and size are proportional to their degree of connectivity; **(G)** chemical structures of metabolites acting on the hub genes.

A total of 154 genes associated with gut microbiota-derived metabolites were curated from gutMGene, and 168 AR-related genes (Relevance score > 10) were retrieved from GeneCards. Intersecting these sets yielded 20 shared genes (Figure 2A): IL4R, IL10, CXCL8, TNF, IL6, ICAM1, IL1B, IL17A, FOXP3, TLR4, TLR2, CCL2, IL12B, CD14, ADRB2, NFKB1, IL22, TLR9, NLRP3, and CYP3A4. These genes corresponded to 9 gut microbiota-derived metabolites, which were enriched in pathways including gut-liver indole metabolism, conjugation of phenylacetate with glutamine, FMO oxidizes nucleophiles, diet-dependent trimethylamine/trimethylamine N-oxide (TMA/TMAO) metabolism, tryptophan catabolism, and amino acid conjugation as well as phenylalanine/tyrosine metabolism (Figure 2B; Supplementary Table 1). Only pathways with *p* < 0.05 are shown, though results may lack significance after FDR testing, requiring cautious interpretation.

### 3.2 GO enrichment analysis and hub gene selection for AR–gut metabolite shared genes

GO enrichment analysis revealed that the 20 shared genes converge on key immune-related functions (*p* < 0.05; Figures 2C, E). The primary biological processes were dominated by the host response to bacterial molecules and the regulation of inflammation. Consistent with a role in immune signaling, the gene products were primarily located on the external side of the plasma membrane and were functionally enriched in cytokine activity and receptor binding. A subsequent Protein-Protein Interaction (PPI) network analysis identified the pro-inflammatory cytokines IL6, TNF, and IL1B as central hub proteins (Figure 2F; Supplementary Table 2). These hubs were linked to the gut microbiota-derived metabolites 3-indolepropionic acid (IPA), succinate, and trimethylamine N-oxide (TMAO). The molecular structures of these metabolites are shown in Figure 2G.

### 3.3 SMR analysis implicates the IL and TLR families as key contributors in AR

Systematic screening of the shared genes showed that many candidates fall within the interleukin (IL) and toll-like receptor (TLR) families, indicating central roles for these gene groups in the development and progression of AR.

Within the network linking gut microbiota metabolites to AR, IL4R emerged as a prominent signal. SMR analysis supported a positive association consistent with causality (top SNP rs8052962; *b*SMR = 0.32; *p*SMR = 0.02). IL4R, located on chromosome 16, is pivotal in immune regulation and Th2 responses and may contribute to AR pathobiology. The HEIDI test yielded *p*HEIDI = 0.99, indicating no evidence of heterogeneity driven by linkage disequilibrium and supporting a potential causal relationship between IL4R expression and the AR phenotype (Supplementary Figure 1; Supplementary Table 3). These findings nominate IL4R as a hub gene.

**Figure 3:**
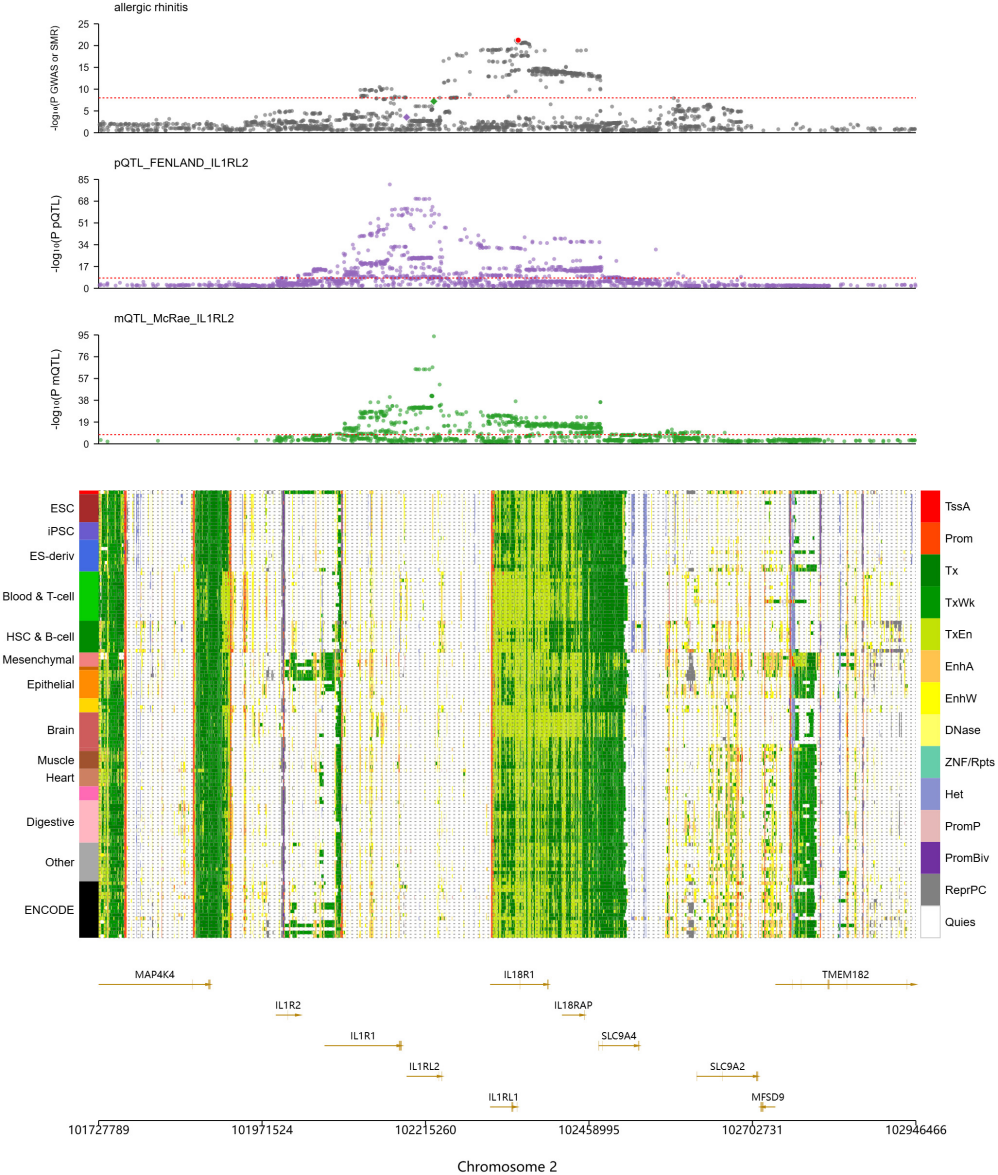
SMR analysis indicates significant genetic associations at IL1RL2 with AR-related DNA methylation and protein expression. The top panel shows colocalization of allergic rhinitis (AR) genome-wide association study (GWAS) signals with IL1RL2-related protein and methylation QTLs (pQTLs/mQTLs) across the gene cluster. The middle panel lists tissue and cell types on the left (e.g., ESC, embryonic stem cells; iPSC, induced pluripotent stem cells) and, on the right, a color legend for chromatin states, including promoter, enhancer, transcriptionally active, heterochromatin, and quiescent states. The bottom panel provides gene annotations and genomic coordinates (including IL1RL1, IL1RL2, IL1R1, IL18R1, and IL18RAP) to pinpoint candidate functional regions.

Other IL family members also showed close connections to AR. As illustrated in Figure 3, several genes on chromosome 2, including IL1RL2, IL1R2, IL1R1, IL18R1, and IL18RAP, align with a region that carries significant association signals in AR GWAS, with the IL1RL2 locus being particularly notable. In mQTL_McRae data, a variant at chr2:102,227,650 was strongly associated with DNA methylation (–log10(mSMR) = 7.2), suggesting genetically regulated methylation at this locus. In the pQTL_FENLAND resource, chr2:102,187,006 was associated with the phenotype (–log10(pQTL-SMR) = 3.5), indicating potential effects at the protein level. Chromatin-state annotations further showed active transcription (Tx) or weak transcription (TxWk) states in immune-relevant cell types such as Blood & T cell and hematopoietic stem cell and B cell, implying transcriptional activity in immune cells and a role in immune regulation.

The TLR1 locus also displayed significant associations with AR. Signals were observed at chr4:38823747 (–log10(eSMR) = 1.26), chr4:38798898 (–log10(sSMR) = 13.84), and chr4:38790677 (–log10(pQTL-SMR) = 8.27), indicating effects at the levels of gene expression, splicing, and protein abundance, with especially strong signals for splicing and protein. Chromatin annotations revealed predominantly Tx states in Blood & T cell, whereas epithelial cells exhibited active enhancer (EnhA) states at the TLR1 region. Because epithelial tissues form the first barrier to exogenous antigens, these regulatory features suggest that TLR1 participates in mucosal immune responses through recognition of pathogen-associated molecular patterns (PAMPs).

### 3.4 Core genes are broadly upregulated in AR tissues

**Figure 4:**
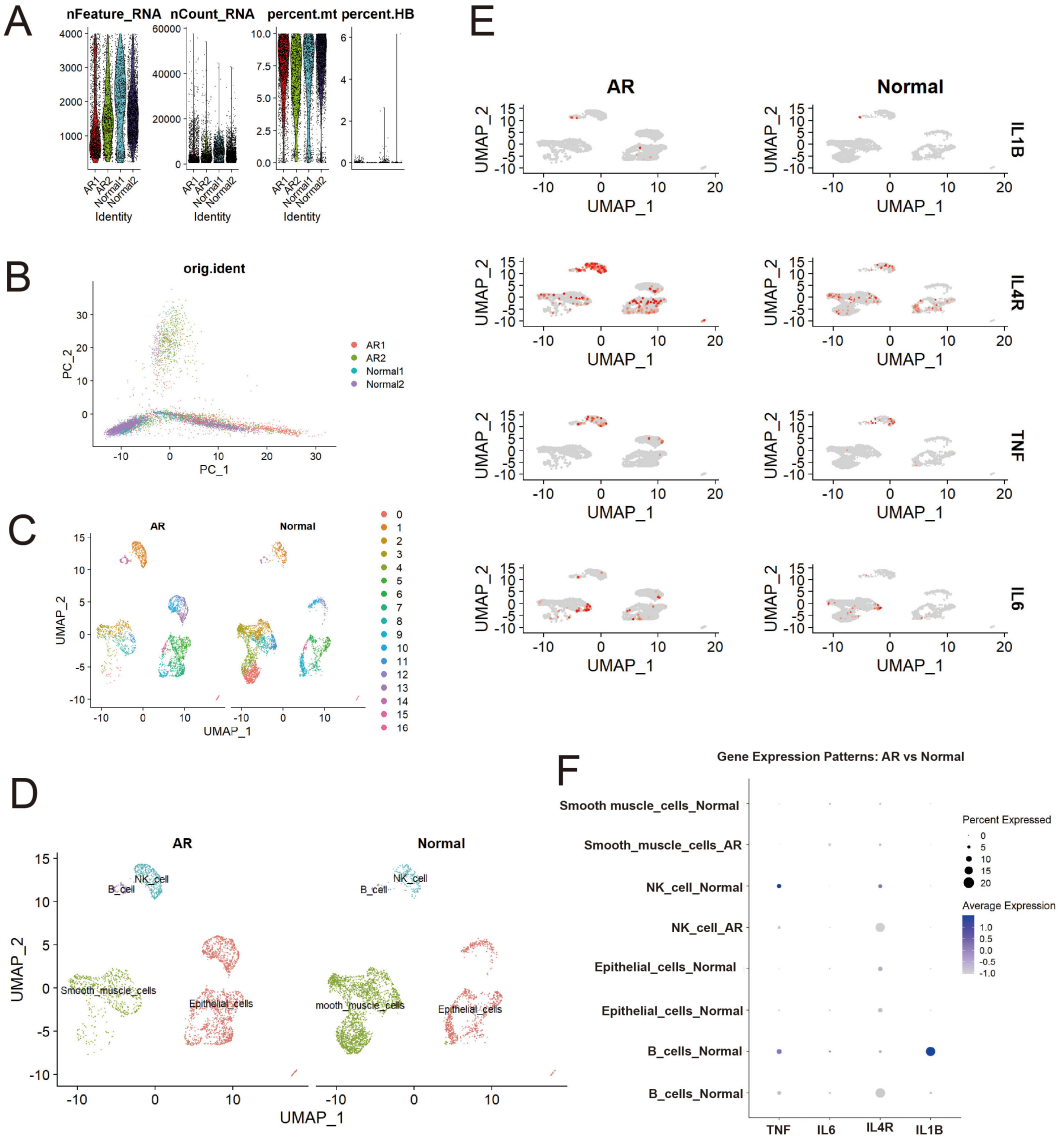
Differential distribution of core genes (IL1B, IL4R, TNF, IL6) between patients with AR and healthy controls. **(A)** Quality control metrics; **(B)** PCA visualization; **(C)** Initial cell clustering; **(D)** Cell annotation results; **(E)** Higher expression of core genes (IL1B, IL4R, TNF, IL6) in AR than in controls; **(F)** Differential expression of core genes across cell types in AR vs. controls.

Single-cell RNA sequencing of AR and normal nasal mucosa revealed distinct cellular composition and transcriptional profiles. Stringent quality control retained cells with appropriate gene counts and low mitochondrial content (Figure 4A), and principal component analysis indicated that batch effects were effectively mitigated (Figure 4B). UMAP embedding separated cells into 17 subclusters (Figure 4C), which were annotated into major lineages including epithelial cells, smooth muscle cells, natural killer (NK) cells, and B cells (Figure 4D). Comparative UMAP distributions showed altered cell-type proportions in AR, with an increased fraction of smooth muscle cells consistent with tissue remodeling and mucosal thickening, alongside expansions of NK and B cells that reflect activated immune and inflammatory responses.

Analysis of our four core candidate genes selected either as high-degree hub genes from the PPI network analysis (IL1B, TNF, and IL6) or for their significant causal link to AR in the SMR analysis (IL4R) demonstrated widespread upregulation in AR. UMAP feature overlays (Figure 4E; Supplementary Table 4) showed more high-expression cells in AR than in controls, with IL4R displaying the most pervasive elevation across the embedding, whereas IL1B, TNF, and IL6 exhibited cluster-restricted peaks.

This dot plot (Figure 4F) reveals significant gene expression shifts in immune cells during AR compared to normal controls. Notably, the expression of pro-inflammatory cytokines IL1B in B cells and TNF in NK cells, present in the normal state, was markedly downregulated in AR. In contrast, the expression pattern of IL4R, a key receptor in type 2 allergic responses, was altered: it became newly expressed in B cells and was found in a higher percentage of NK cells during AR, despite a lower average expression level. These findings suggest a suppression of specific pro-inflammatory pathways and a concurrent modulation of allergy-related signaling in B cells and NK cells in the context of Allergic Rhinitis.

### 3.5 Shared target-driven candidate therapies for AR

Integration of the PPI network, SMR, and single-cell analyses identified IL6, TNF, IL1B, and IL4R as core pathogenic genes in AR. Upstream gut microbiota-derived metabolites modulating these genes were mined from gutMGene, yielding IPA, succinate, TMAO, and butyrate. Mechanistic links between these metabolites and the core genes were supported by prior studies: IPA, a tryptophan-derived microbial metabolite, suppresses NF-κB signaling and lowers IL6 levels (Zhao et al., 2019); succinate, in concert with TLR ligands, induces dendritic cells (DCs) to produce TNF (Rubic et al., 2008); TMAO markedly upregulates IL1B expression (Meng et al., 2019); and butyrate promotes IL4R expression (Pearce et al., 2020).

**Figure 5:**
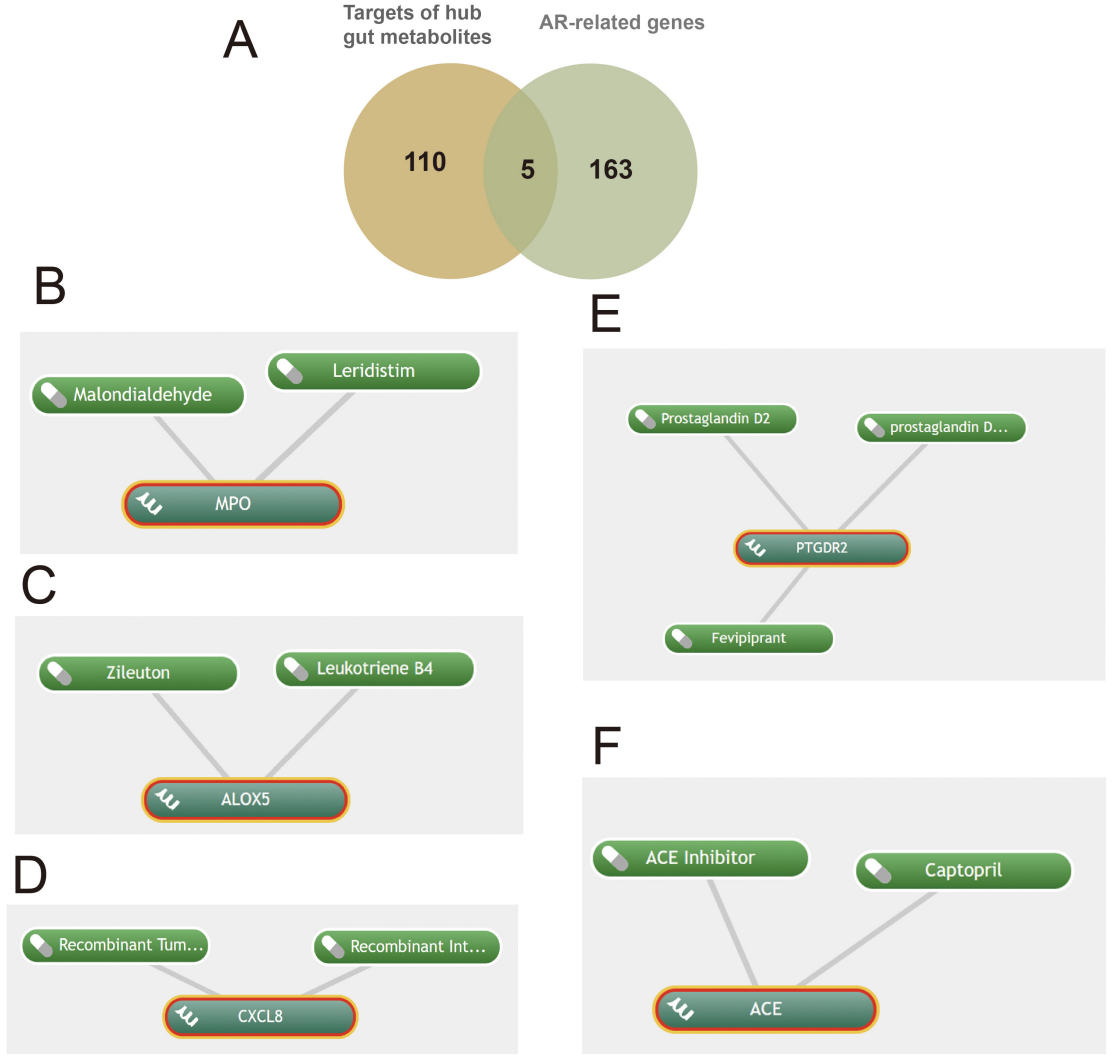
Shared targets in allergic rhinitis (AR) linked to gut microbiota-derived metabolites with predicted drugs. **(A)** Five shared targets; **(B-F)** drugs identified via Coremine targeting MPO, ALOX5, CXCL8, PTGDR2, and ACE.

Target prediction for these metabolites followed by intersection with the AR-related gene set (Figure 5A) yielded five key targets—MPO, PTGDR2, ALOX5, ACE, and CXCL8—and enabled prioritization of candidate drugs (Figures 5B–F). Clinical status and annotations were cross-checked in DrugBank and FDA records (Table 2). Fevipiprant, which targets PTGDR2, has been evaluated in clinical trials for asthma and atopic dermatitis; Zileuton, a 5-lipoxygenase inhibitor that reduces leukotriene synthesis, targets ALOX5; and captopril, an angiotensin-converting enzyme inhibitor, targets ACE. These agents exhibit translational potential for AR. By contrast, malondialdehyde (MDA) is typically used as a biomarker of oxidative damage rather than a therapeutic, and leukotriene B4 (LTB4) is generally considered an inflammatory mediator or biomarker.

**Table 2:** Detailed Information of Drugs/Molecules Targeting MPO, PTGDR2, ALOX5, ACE, and CXCL8.

**Target**	**Predicted Drug/Molecule**	**PubChem CID**	**Molecular Formula**	**Description**
MPO	Malondialdehyde	10964	C3H4O2	Malondialdehyde is a biomarker of lipid oxidative damage caused by smoking, mainly existing in the enol form *in vivo*.
PTGDR2	Fevipiprant	23582412	C19H17F3N2O4S	Fevipiprant has been used in trials studying the treatment of Asthma, Atopic Dermatitis, and Allergic Rhinitis. (DrugBank)
	Prostaglandin D2	448457	C20H32O5	NA
ALOX5	Zileuton	60490	C_11_H_12_N_2_O_2_S	Zileuton is a non-steroidal anti-inflammatory drug, anti-asthmatic agent, leukotriene antagonist, and ferroptosis inhibitor. (ChEBI)
	Leukotriene B4	5280492	C_20_H_32_O_4_	Acts as a human, mouse, and plant metabolite, and functions as a vasoconstrictor agent. (ChEBI)
ACE	Captopril	44093	C9H15NO3S	Captopril is an angiotensin-converting enzyme (ACE) inhibitor. (FDA)
	ACE Inhibitor	37056	C53H76N14O12	NA
CXCL8	Recombinant Interleukin-6	NA	NA	NA

The table includes PubChem Compound Identifiers (CID), molecular formulas, and brief descriptions of each compound's biological role or clinical relevance. Abbreviations: MPO (Myeloperoxidase), PTGDR2 (Prostaglandin D2 receptor 2), ALOX5 (Arachidonate 5-lipoxygenase), ACE (Angiotensin-converting enzyme), CXCL8 (C-X-C motif chemokine ligand 8), NA (Not available), ChEBI (Chemical Entities of Biological Interest), FDA (U.S. Food and Drug Administration).

### 3.6 Molecular docking and ADMET assessment of predicted compounds

**Figure 6:**
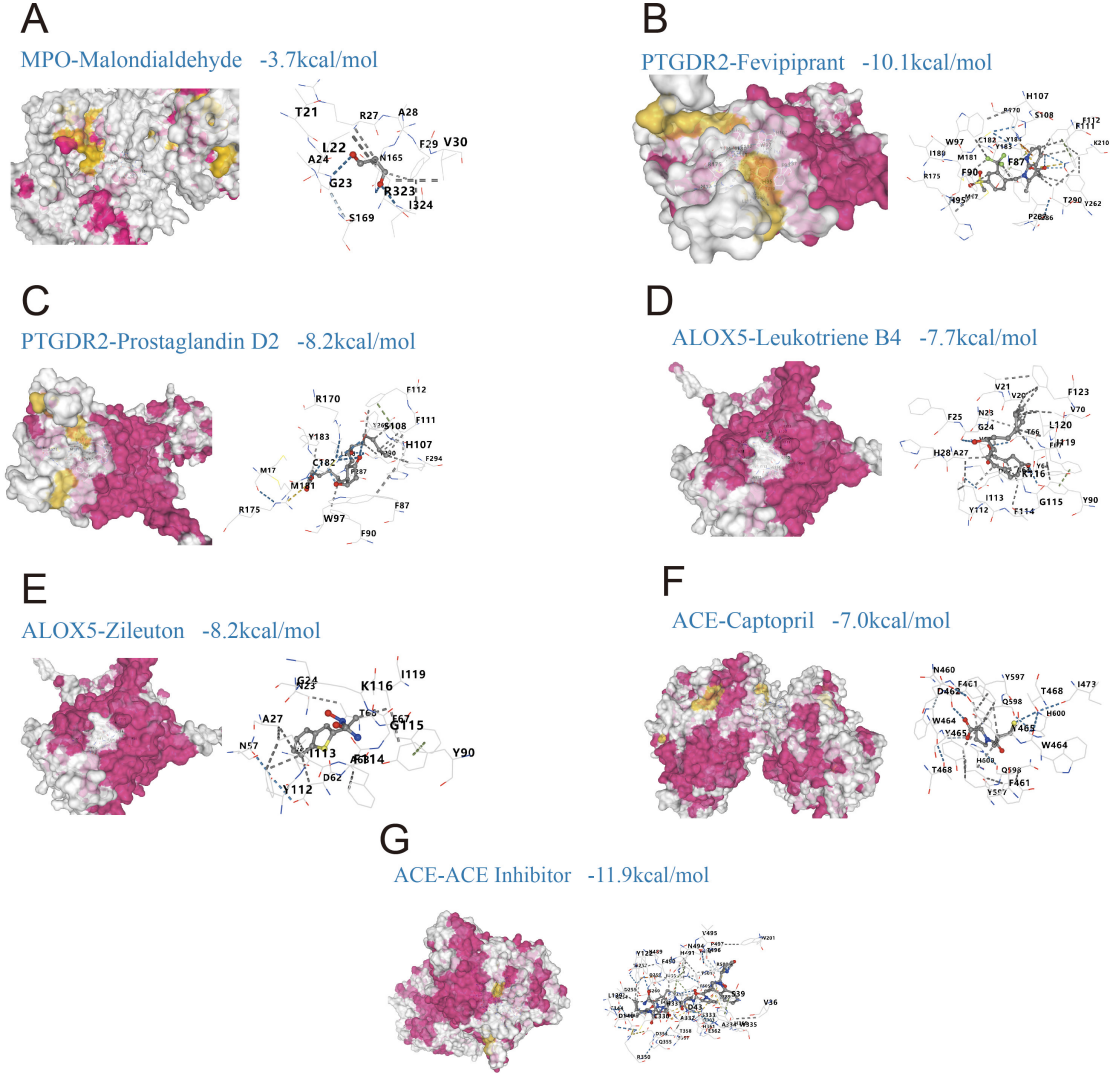
Molecular docking of predicted drugs with shared targets linking gut microbiota-derived metabolites and allergic rhinitis (AR). **(A)** MPO with malondialdehyde, binding energy −3.7 kcal/mol; **(B)** PTGDR2 with Fevipiprant, −10.1 kcal/mol; **(C)** PTGDR2 with prostaglandin D2, −8.2 kcal/mol; **(D)** ALOX5 with leukotriene B4, −7.7 kcal/mol; **(E)** ALOX5 with Zileuton, −8.2 kcal/mol; **(F)** ACE with captopril, −7.0 kcal/mol; **(G)** ACE with an ACE inhibitor, −11.9 kcal/mol.

Molecular docking showed that most compound-target complexes achieved binding energies at or below −7.0 kcal/mol, indicating favorable binding under the study criteria. The strongest affinities were observed for PTGDR2 with Fevipiprant, −10.1 kcal/mol (Figure 6B), and for ACE with an ACE inhibitor, −11.9 kcal/mol (Figure 6G). Additional pairs also performed well: PTGDR2 with prostaglandin D2, −8.2 kcal/mol (Figure 6C); ALOX5 with leukotriene B4, −7.7 kcal/mol (Figure 6D); ALOX5 with Zileuton, −8.2 kcal/mol (Figure 6E); and ACE with captopril, −7.0 kcal/mol (Figure 6F). By contrast, MPO with malondialdehyde (MDA) yielded −3.7 kcal/mol (Figure 6A), which did not meet the feasibility threshold defined in the Methods (≤ −5.0 kcal/mol). Pocket analyses indicated stable contacts with key residues, including W97, H107, and F87 in the complex of PTGDR2 with Fevipiprant (Figure 6B) and K116, G115, and Y112 in the complex of ALOX5 with Zileuton (Figure 6E).

ADMET profiling using ADMETlab 3.0 supported generally favorable drug-likeness according to Lipinski's rule of five (Table 3). Zileuton (MW 236.29, TPSA 94.8, logP 1.6, HBD 2, HBA 3) and captopril (MW 217.29, TPSA 58.6, logP 0.3, HBD 2, HBA 4) satisfied all criteria, and prostaglandin D2 (MW 352.5, TPSA 94.8, logP 2.6, HBD 3, HBA 5) largely conformed as well. The generic ACE inhibitor used for docking (MW 1,101.3, TPSA 387, logP −0.9, HBD 10, HBA 13) exceeded multiple thresholds, indicating potential challenges for oral bioavailability. Together, the docking and ADMET results support strong target engagement for several candidates alongside acceptable developability profiles, with exceptions for very large ligands.

**Table 3:** Lipinski's Rule of Five evaluation results for predicted drugs.

**Compound**	**MW**	**TPSA**	**Log *P***	**HBD**	**HBA**
Fevipiprant	426.4	97.6	2.8	1	8
Prostaglandin D2	352.5	94.8	2.6	3	5
Zileuton	236.29	94.8	1.6	2	3
Captopril	217.29	58.6	0.3	2	4
ACE Inhibitor	1101.3	387	−0.9	10	13

MW, molecular weight; (<500 g/mol); TPSA, topological polar surface area; (<140 Å^2^); Log P, logarithm of the partition coefficient; (<5); HBA, hydrogen bond acceptor; (<10); HBD, hydrogen bond donor; (<5).

## 4 Discussion

The gut-nose axis, as a crucial mechanism in mucosal immune regulation, is emerging as a research frontier in allergic diseases, though its specific mechanisms remain poorly understood. Current research widely suggests that the gut microbiota's influence on nasal mucosal tissue is primarily mediated through systemic circulation. By fermenting substrates like dietary fiber and protein, the gut microbiota produces various metabolites, such as SCFAs, secondary bile acids, tryptophan metabolites, and lipopolysaccharide (LPS). These metabolites enter the systemic circulation via the intestinal mucosa and act on distant tissues, including the nasal mucosa (Tai et al., 2021). Studies have confirmed that nasal and airway epithelial cells express the SCFA receptors GPR41 and GPR43, and one study demonstrated that SCFAs can activate these receptors to induce tissue plasminogen activator (t-PA) expression, holding therapeutic potential for reducing fibrin deposition in nasal polyps (Wu et al., 2024). Conversely, local immune changes in the nasal mucosa can also influence the gut microbiota, indicating a bidirectional regulation. Immunoglobulin A (IgA), a key player in mucosal immunity, is produced in both the nasal mucosa and the intestinal lamina propria. IgA from the nasal mucosa can reach the gut via circulation, and its deficiency can lead to gut dysbiosis, persistent immune activation, and inflammation (Fagarasan and Honjo, 2004). An experimental study further confirmed this link, finding that honeysuckle polysaccharides improved nasal inflammation by inhibiting the NLRP3 inflammasome and IL-17, while also reducing NLRP3 in the gut to maintain microbiota homeostasis (Bai et al., 2022). Building on this foundation, we investigated the downstream genes regulated by gut microbiota metabolites that are involved in the pathological process of AR. Through a multi-faceted approach combining tissue correlation, causal inference (SMR), single-cell analysis, and drug screening, we identified candidate core genes potentially linking gut metabolites to AR and proposed potential therapeutic agents targeting them.

Twenty microbiota-derived metabolites were linked to AR-related genes. The shared genes were enriched for responses to bacterial molecules, cytokine signaling, chemotaxis, and membrane receptor—mediated immune recognition, implicating them in AR pathogenesis. The upstream metabolites mapped predominantly to tryptophan catabolism, conjugation of phenylacetate with glutamine, and diet-dependent TMA/TMAO metabolism. Tryptophan-derived indoles can activate the AhR, modulate cytokines such as IL-22 and IL-17A, maintain nasal mucosal barrier integrity, and temper local inflammatory responses. By contrast, phenylacetate–glutamine conjugates and TMAO may promote systemic inflammation, potentially by enhancing the NLRP3 inflammasome and pattern-recognition receptors including TLR2 and TLR4, thereby fueling gut-nasal axis–mediated chronic allergic inflammation (Liu et al., 2020). In line with the concept of metabolic intervention, alanyl-glutamine has been reported to reshape the gut microbiota, increase butyrate, activate AMP-activated protein kinase (AMPK) signaling, and inhibit the NLRP3 inflammasome, thereby alleviating inflammatory pathology in allergic asthma (Liu et al., 2020). These observations suggest that targeting glutamine metabolism (for example, inhibiting glutaminase) or blocking TMAO production could offer therapeutic strategies for chronic atopic disease. Dysregulated tryptophan metabolism is also pertinent to AR pathogenesis. Dysbiosis can reduce indole derivatives, weakening AhR-dependent barrier maintenance and predisposing to the systemic spread of allergic inflammation. In a food allergy mouse model, disruption of the tryptophan pathway suppressed Reg3g and IL-22 expression and disturbed intestinal immune homeostasis (Wang et al., 2024). Another study showed that the prebiotic fructo-oligosaccharide (FOS) modulated the gut microbiota and tryptophan metabolism, increased indole-3-acetate (IAA) and IPA, activated the AhR/IL-22 axis, and improved a DSS-induced allergic-like phenotype in mice (Yang et al., 2024).

**Figure 7:**
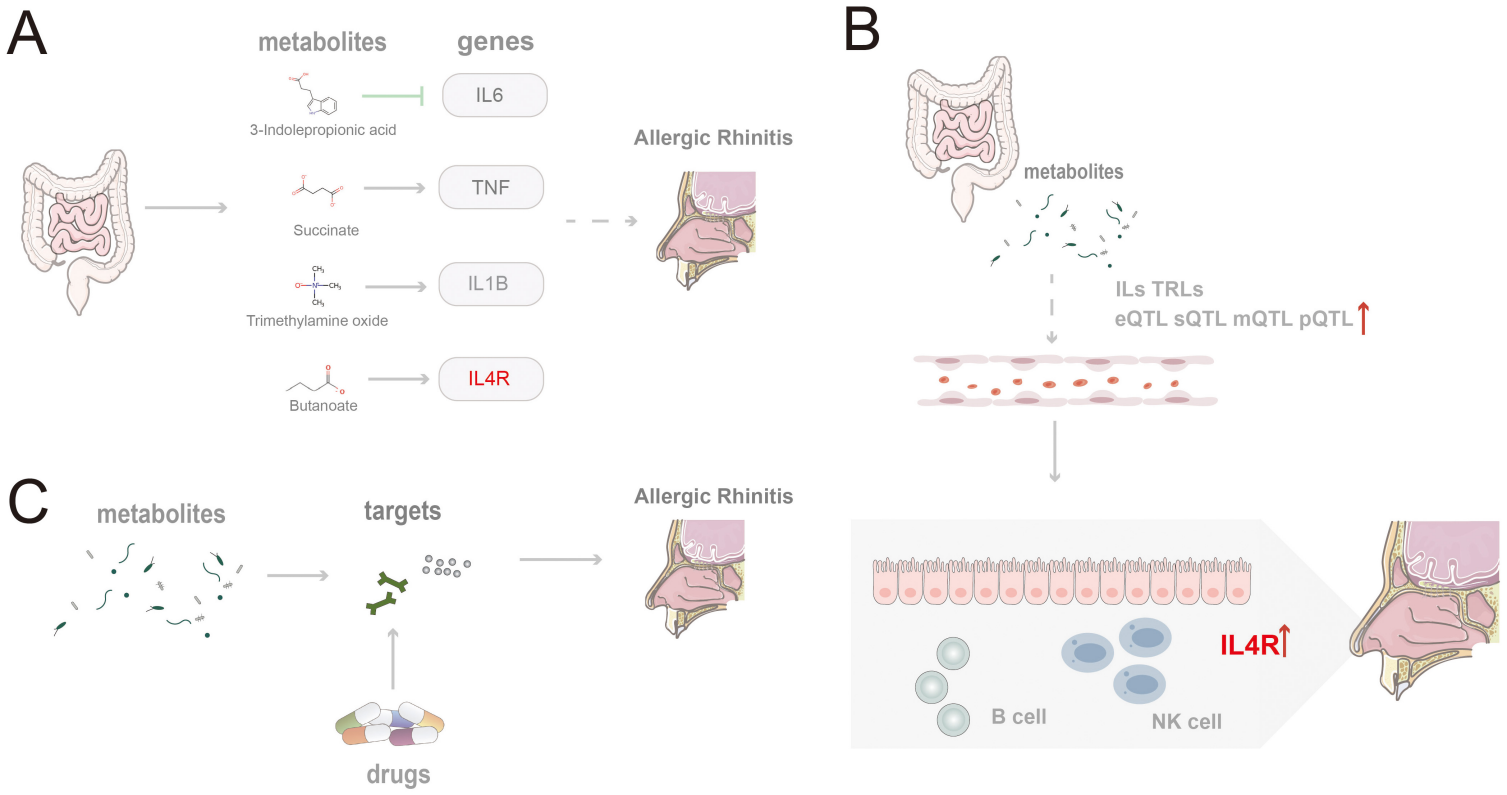
Schematic summary of the study findings. **(A)** Four gut microbiota-derived metabolites (indole-3-propionic acid, succinate, trimethylamine N-oxide, and butyrate) modulate core inflammatory genes and promote AR pathogenesis; **(B)** Microbiota-derived metabolites shape epigenetic regulation and expression of IL and TLR family genes, thereby facilitating AR; **(C)** Candidate therapeutics act on targets shared by gut metabolites and AR.

Our analysis identified associations between several gut microbiota metabolites—namely, 3-IPA, succinate, TMAO, and butyrate—modulate the progression of AR by regulating their corresponding target genes: IL6, TNF, IL1B, and IL4R (Figure 7A). As an indole derivative of tryptophan metabolism, 3-IPA exhibits a distinct protective role. It alleviates mucosal inflammation by downregulating IL-6 via inhibition of the NF-κB signaling pathway (Zhao et al., 2019; Peng et al., 2019). Furthermore, it enhances epithelial barrier stability by activating the AhR/IL-22 axis (Zelante et al., 2013) and demonstrates potent anti-inflammatory properties in animal models by inhibiting Th2-related cytokines and IgE levels while upregulating IL-10 expression through the AKT/CEBPβ pathway (Gao et al., 2025). Other studies have indicated that AR patients exhibit dysregulation in tryptophan and fatty acid metabolism within their nasal microbiome and serum metabolome, with an imbalance in n-3 polyunsaturated fatty acids being significantly associated with AR risk (Yuan et al., 2021; Nakamura et al., 2025). As an inflammatory cytokine regulated by fatty acid metabolism, IL-6 may indirectly mediate the immunological consequences of this metabolic dysregulation. Beyond its regulation by tryptophan metabolism, IL-6 is also modulated by SCFAs. For instance, SCFAs like butyrate can regulate the Th17/Treg balance and downregulate IL-6 expression, thereby mitigating mucosal inflammation (Yang et al., 2024; Hou et al., 2024).

Succinate, as an intermediate metabolite of the TCA cycle, exhibits pro-inflammatory effects. It can induce TNF expression and promote Th2 polarization by stabilizing HIF-1α and synergizing with TLR ligands to activate dendritic cells (Tannahill et al., 2013; Rubic et al., 2008), potentially exacerbating the inflammatory response in AR. TNF is a key factor in the initiation and amplification of the early inflammatory response in AR, and its expression is significantly modulated by gut microbiota metabolites. In a PM2.5-induced AR mouse model, the upregulation of TNF-α was accompanied by the activation of the NLRP3 inflammasome and gut dysbiosis (Li et al., 2025). Furthermore, tryptophan metabolites such as kynurenine can inhibit TNF-α expression and modulate the Th1/Th2 balance via the AhR signaling pathway (Xu et al., 2022; Lau et al., 2021). Intervention combining probiotics and prebiotics has also been shown to ameliorate clinical symptoms by modulating TNF signaling (Xu et al., 2022; Lau et al., 2021).

TMAO, generated from the microbial metabolism of choline and carnitine, has been shown to induce IL1B expression in monocytes and activate pro-inflammatory pathways, potentially promoting persistent local inflammation in the nasal mucosa in the setting of AR (Meng et al., 2019; Liu et al., 2020). IL-1β, encoded by *IL1B*, acts as a key amplifying factor linking microbial metabolic dysregulation to the inflammation of chronic rhinitis. It exacerbates nasal mucosal damage by enhancing Th2/Th17 polarization and eosinophil infiltration. In AR models, exposure to PM2.5 induces activation of the NLRP3–IL-1β axis, accompanied by the enrichment of Ileibacterium and Alistipes, and dysregulated bile acid metabolism (Li et al., 2025; Theiler et al., 2019). Conversely, SCFAs can effectively reduce IL-1β levels by inhibiting HDAC and NF-κB signaling (Pedersen et al., 2022). Furthermore, multiple genes within the IL and TLR families show a significant epigenetic association with AR (Figure 7B).

Butyrate is generated from the fermentation of dietary fiber by gut microbiota. It promotes IL-10 expression and Treg cell differentiation by inhibiting HDAC activity (Furusawa et al., 2013) while also potentially upregulating IL4R expression to enhance Th2-type responses (Pearce et al., 2020). SMR analysis revealed a significant positive causal relationship between *IL4R* (topSNP: rs8052962) and AR (bSMR = 0.32, *p*SMR = 0.02). Located on chromosome 16, IL4R is the core receptor for the Th2-type immune response, and its expression is significantly modulated by the AhR pathway. Tryptophan metabolites, such as IAA and 3-IAld, can activate AhR, which in turn induces IL-22 production, enhances intestinal barrier function, and inhibits Th2 polarization, thereby indirectly suppressing IL4R-mediated immune responses (Pei et al., 2025; Fang et al., 2022; Kou et al., 2024). Furthermore, the expression level of IL4R is positively correlated with IgE memory B cells (Aranda et al., 2023).

To facilitate clinical translation, five potential therapeutic targets shared by gut microbiota-derived metabolites and AR—MPO, PTGDR2, ALOX5, CXCL8, and ACE—were prioritized, and drugs acting on these targets were mined (Figure 7C). Fevipiprant targets PTGDR2 and showed a binding energy of −10.1 kcal/mol with a stable pose. PTGDR2 is activated by prostaglandin D2 and mediates proinflammatory chemotaxis of eosinophils, basophils, and Th2 lymphocytes, playing a key role during allergic inflammation. Its antagonist Fevipiprant has entered clinical studies in Th2-type allergic diseases such as asthma, and a randomized, double-blind, controlled trial reported reduced eosinophilic airway inflammation with good tolerability in patients with elevated sputum eosinophils (Gonem et al., 2016). These findings support PTGDR2 as a tractable target and motivate the design of PTGDR2-directed therapies for eosinophilic allergic conditions, including AR. Zileuton, identified for ALOX5, is a 5-lipoxygenase inhibitor within the leukotriene pathway and has been shown to lower eosinophils as well as LTB4, IL-5, IL-6, and TNF-α (Hasday et al., 2000). Current evidence supports its use in asthma (Thalanayar Muthukrishnan et al., 2020), and the present results suggest potential repurposing for AR. Most of the highlighted agents satisfied Lipinski's rule-of-five filters, indicating favorable oral developability in addition to therapeutic promise.

Notably, among the screened molecules, LTB4 and Malondialdehyde (MDA) are more appropriately considered biomarkers rather than interventions. LTB4 is synthesized from arachidonic acid in myeloid cells via 5-lipoxygenase and LTA4 hydrolase, can drive antimicrobial peptide production through PI3K and Src signaling, interacts with TLR pathways to enhance cytokine production, and modulates innate immunity; serum LTB4 is typically higher in patients with AR than in healthy controls (Ghada et al., 2018). MDA is a terminal product of membrane lipid peroxidation and serves as a marker of oxidative damage and inflammation in AR; agents such as N-acetylcysteine (NAC) and resveratrol can reduce MDA formation and alleviate allergic inflammation (Zhang et al., 2020).

Several limitations of this study should be acknowledged. First, the small sample size used for our single-cell analysis is a notable constraint. Although we identified genes associated with gut metabolites and Allergic Rhinitis, the scarcity of public data for relevant tissues precluded a deeper investigation into potential mediating effects, a gap that warrants future experimental validation. Furthermore, while Fevipiprant and Zileuton were identified as potential therapeutic candidates, their clinical efficacy and optimal therapeutic strategies must be established through rigorous clinical trials. This includes determining whether Zileuton should be used in combination with other anti-inflammatory agents, such as inhaled corticosteroids, for AR treatment (Mastalerz and Kumik, 2010), and defining the precise role of Fevipiprant as either a monotherapy or an adjuvant therapy (Issahaku et al., 2019). Dietary patterns, antibiotic exposure, and lifestyle factors that shape the microbiota and its metabolites were not fully captured and may indirectly influence AR through an individual-microbiota-metabolite-gene regulatory network. Interindividual variability and temporal dynamics of the gut microbiota were also not fully addressed. On the therapeutic side, strategies to supplement or modulate microbiota-derived metabolites (for example, probiotics and prebiotics) and fecal microbiota transplantation (FMT) may offer adjunctive options for AR and warrant further investigation. Evidence suggests that FMT can repair epithelial barriers, rebalance CD4+ T-cell subsets, and exert anti-inflammatory effects via PI3K/AKT/mTOR and NF-κB pathways, thereby alleviating allergic inflammation in AR (Dong et al., 2024). Future work should focus on the gut-nasal axis by integrating metagenomics, metabolomics, and clinical cohort data to evaluate the feasibility of individualized interventions, and by validating key targets and drugs in animal models and organoid systems to advance AR therapy from symptom control toward mechanism-based precision treatment.

In summary, this integrative multi-omics analysis elucidates how gut microbiota-derived metabolites contribute to AR pathogenesis, offering a new perspective on the gut-nasal axis in allergic disease. Despite certain limitations, the findings provide a theoretical basis for precision diagnosis and individualized therapy in AR. Future work should prioritize mechanistic validation, optimization of multi-omics integration, and clinical translation to move the field from association toward causal mechanisms and precision therapeutics.

## 5 Conclusion

This study identified four core genes (IL4R, IL6, IL1B, and TNF) associated with gut microbiota metabolites and AR. It also uncovered potential drugs, Fevipiprant and Zileuton, and biomarkers, MDA and LTB4. However, due to the exploratory nature of this research, these findings should be interpreted with caution. Further validation through *in vitro* cell experiments and animal models is crucial to elucidate the specific effects of these drugs.

## Data Availability

The datasets presented in this study can be found in online repositories. The names of the repository/repositories and accession number(s) can be found in the article/Supplementary material.
