# Supplementary material for: Gut microbiota metabolites and key target molecules in allergic rhinitis: a multi-omics study of gut-nose axis regulation via the inflammation-metabolism network
Source: Front Microbiol. 2025 Nov 27;16:1702518. doi: 10.3389/fmicb.2025.1702518 (PMC12696707; doi:10.3389/fmicb.2025.1702518)
Supplement: Supplementary file 2 [file Data_Sheet_1.docx]

# Supplementary Method

## Functional Annotation and Pathway Analysis using FUMA

To elucidate the biological context of the shared genetic architecture between allergic rhinitis and gut metabolites, we employed the GENE2FUNC module of the FUMA platform (v1.5.x, Ensembl v102) for functional annotation. To mitigate potential confounding from the highly polymorphic Major Histocompatibility Complex (MHC) region, genes within this locus were excluded from all analyses.

The primary objective was to determine the tissue-specific expression patterns of these shared genes. We conducted a tissue specificity analysis using data from the Genotype-Tissue Expression (GTEx) v8 project, encompassing 30 general and 54 specific tissue types. A differentially expressed gene (DEG) set enrichment test, built into FUMA, was used to evaluate whether the shared genes were significantly over-expressed in each tissue relative to all others. The Benjamini-Hochberg (BH) procedure was applied to control for multiple comparisons, with an adjusted P-value < 0.05 being the criterion for significant enrichment.

Following the identification of relevant tissues, we performed gene set enrichment analysis to uncover the underlying molecular functions. Using a hypergeometric test, we assessed the enrichment of our gene list against Gene Ontology (GO) gene sets. The background gene set comprised all annotated genes in Ensembl (e.g., protein-coding, lncRNA, ncRNA). A pathway was considered significantly enriched if it met the criteria of a False Discovery Rate (FDR) < 0.05 and contained at least two of the input shared genes.

# Supplementary Results

## FUMA Analysis Results

***Shared Genes Are Specifically Enriched in Immune and Metabolic Tissues***

To investigate the primary sites of action for the shared genes connecting allergic rhinitis and gut metabolites, we first performed a tissue-specific expression enrichment analysis. A macro-level analysis based on the 30 general tissue types from the GTEx v8 database revealed that the gene set exhibited the most significant expression enrichment in the Lung and Spleen (Supplementary Figure 1). Furthermore, significant enrichment signals were also observed in Adipose Tissue, Colon, and Brain.

To obtain a more precise localization, we further analyzed expression data from 54 specific tissues (Supplementary Figure 2). The results not only confirmed the above findings but also provided more specific information: the expression of shared genes was significantly enriched in the Lung, Spleen, Adipose (Visceral Omentum), and Colon (Transverse). This expression pattern, moving from a general to a specific level, strongly suggests that the core functions of these shared genes involve both respiratory and immune tissues directly related to allergic rhinitis (Lung, Spleen), as well as digestive and metabolic tissues closely associated with gut metabolism (Colon, Adipose Tissue).

***Shared Genes Are Primarily Involved in Immune Recognition and Cytokine Signaling***

Having identified the key tissues of action, we further elucidated the specific roles these genes play at the cellular level through Gene Ontology (GO) molecular function enrichment analysis (Supplementary Figure 3). The results clearly pointed to a core functional module: immune signal recognition and response.

The most significantly enriched molecular functions included "cytokine activity," "cytokine receptor binding," and "signaling receptor binding." Critically, multiple pathways directly related to innate immune recognition were also highly enriched, such as "pattern recognition receptor activity" and "lipopolysaccharide immune receptor activity."

By examining the specific genes involved in these functions (Supplementary Figure 3, right-side heatmap), we found that they were primarily driven by a core set of immune genes. These included Toll-like receptors (TLR2, TLR4) responsible for recognizing microbial products, the key inflammasome protein (NLRP3), and a series of cytokines and chemokines that play pivotal roles in the immune response (e.g., IL1B, IL6, IL10, IL17A, CCL2).


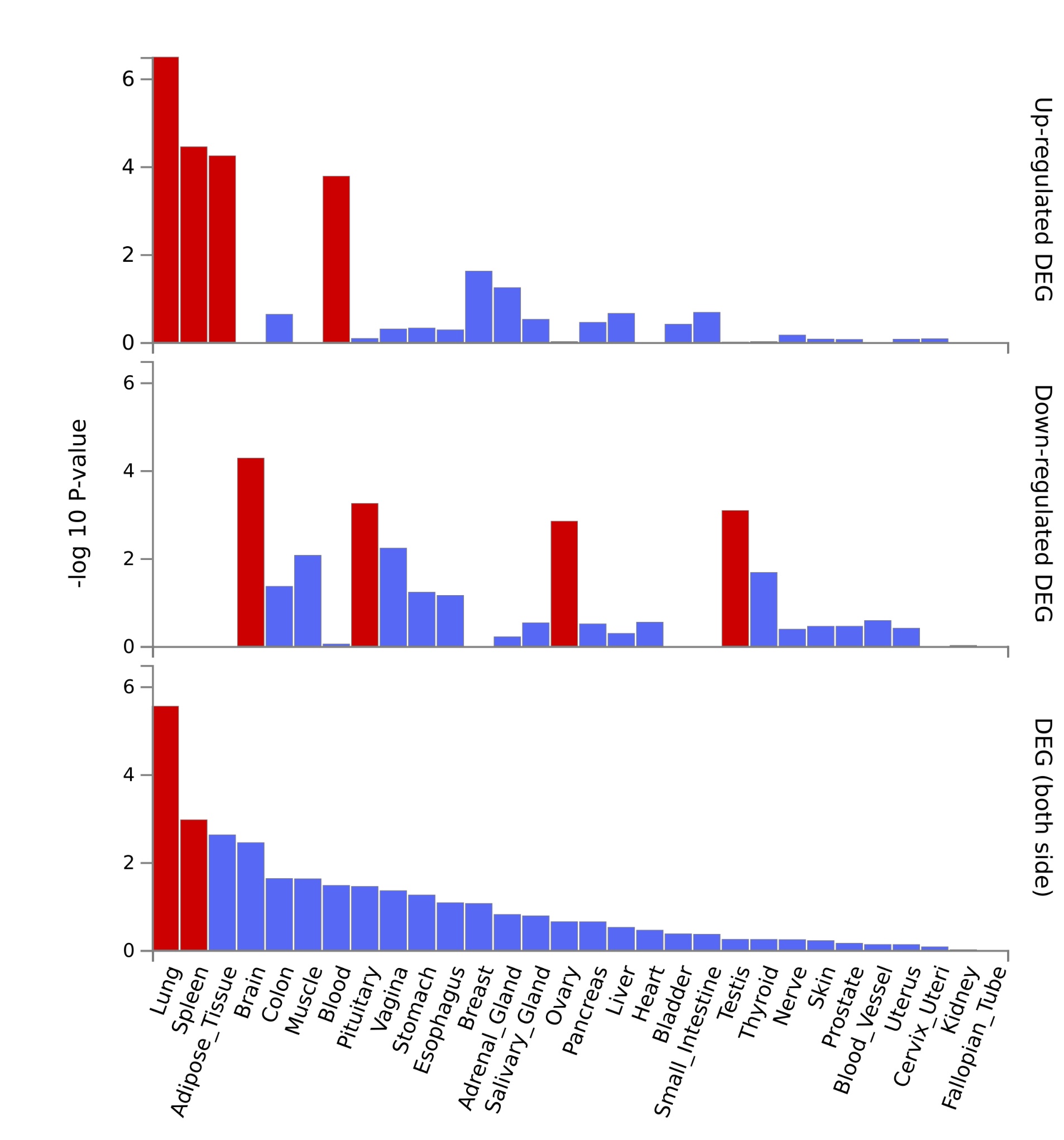


**Supplementary Figure 1.** Expression enrichment analysis of shared genes across 30 general tissue types from the GTEx v8 database.

The plot displays the enrichment results in broad tissue categories. The y-axis represents the -log10 transformed, multiple-testing corrected P-value, and the x-axis lists the different tissues. Taller bars indicate more significant expression enrichment of the shared gene set in the corresponding tissue.


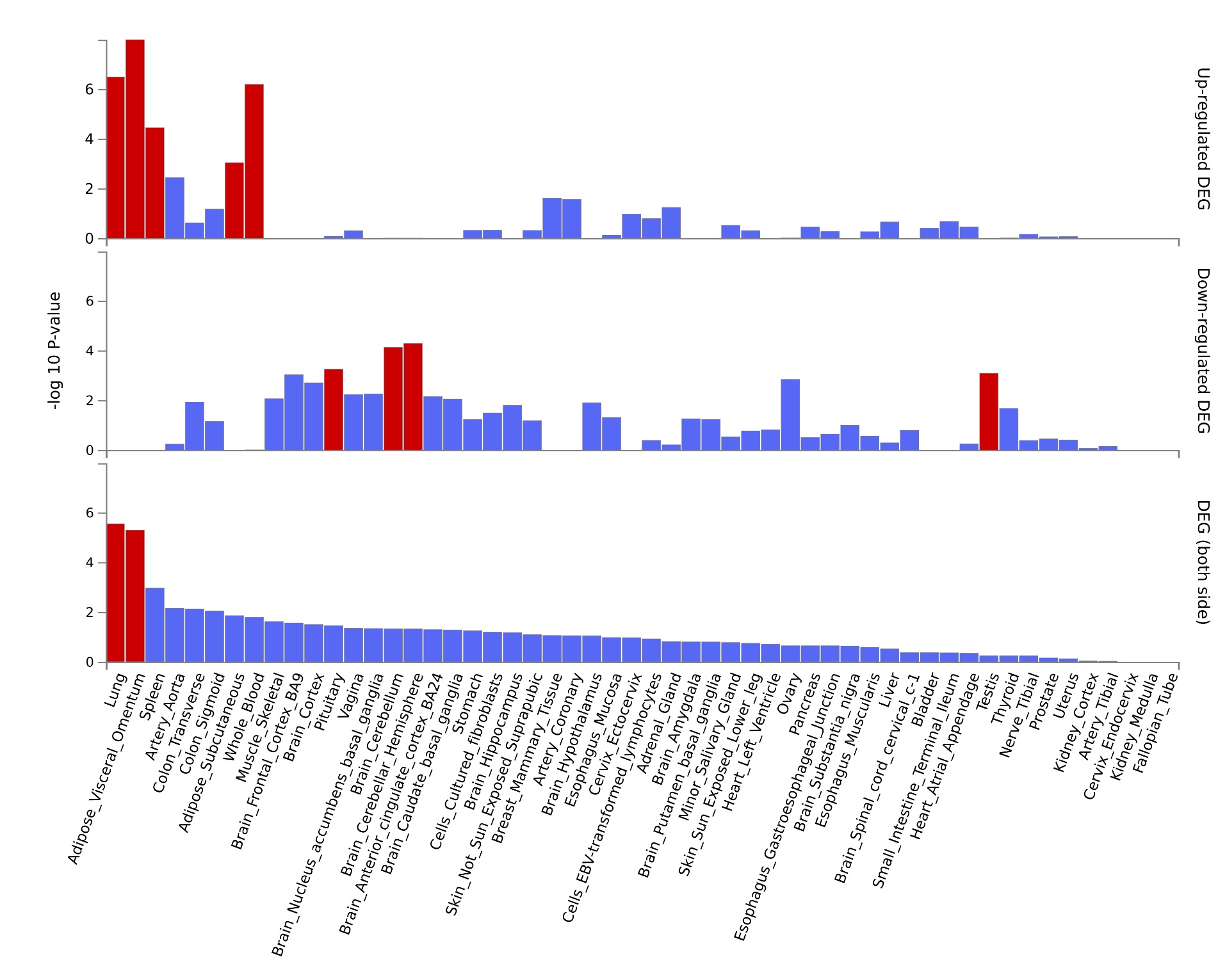


**Supplementary Figure 2**. Expression enrichment analysis of shared genes across 54 specific human tissues from the GTEx v8 database.

This plot provides a more detailed refinement of Supplementary Figure 1, showing enrichment in more specific tissue subtypes. The axes are interpreted in the same way as in Supplementary Figure 1.


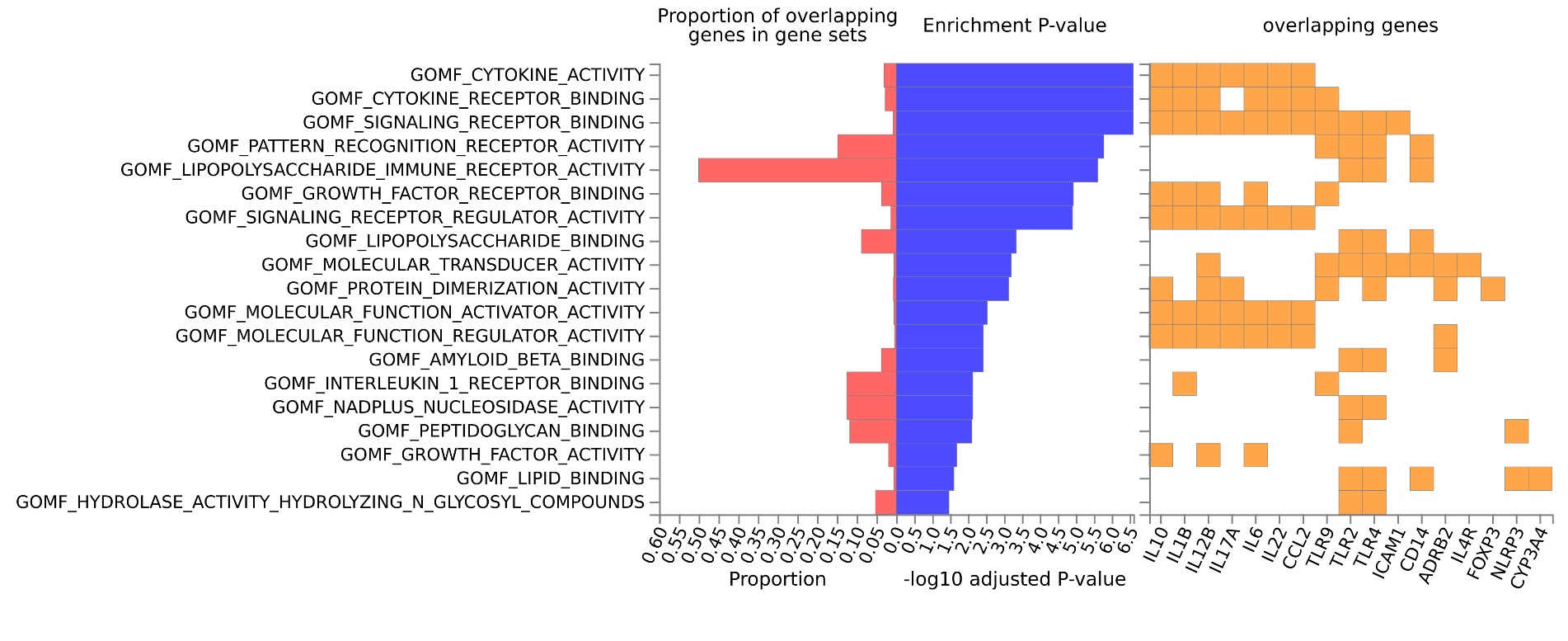


**Supplementary Figure 3.** GO molecular function enrichment analysis of the shared genes.

The bar plot on the left displays the significantly enriched GO molecular function terms; the blue bars represent the enrichment *P*-value, and the red bars represent the proportion of input genes within the corresponding term. The heatmap on the right illustrates the specific shared genes included in each GO term.

## SMR Analysis Results


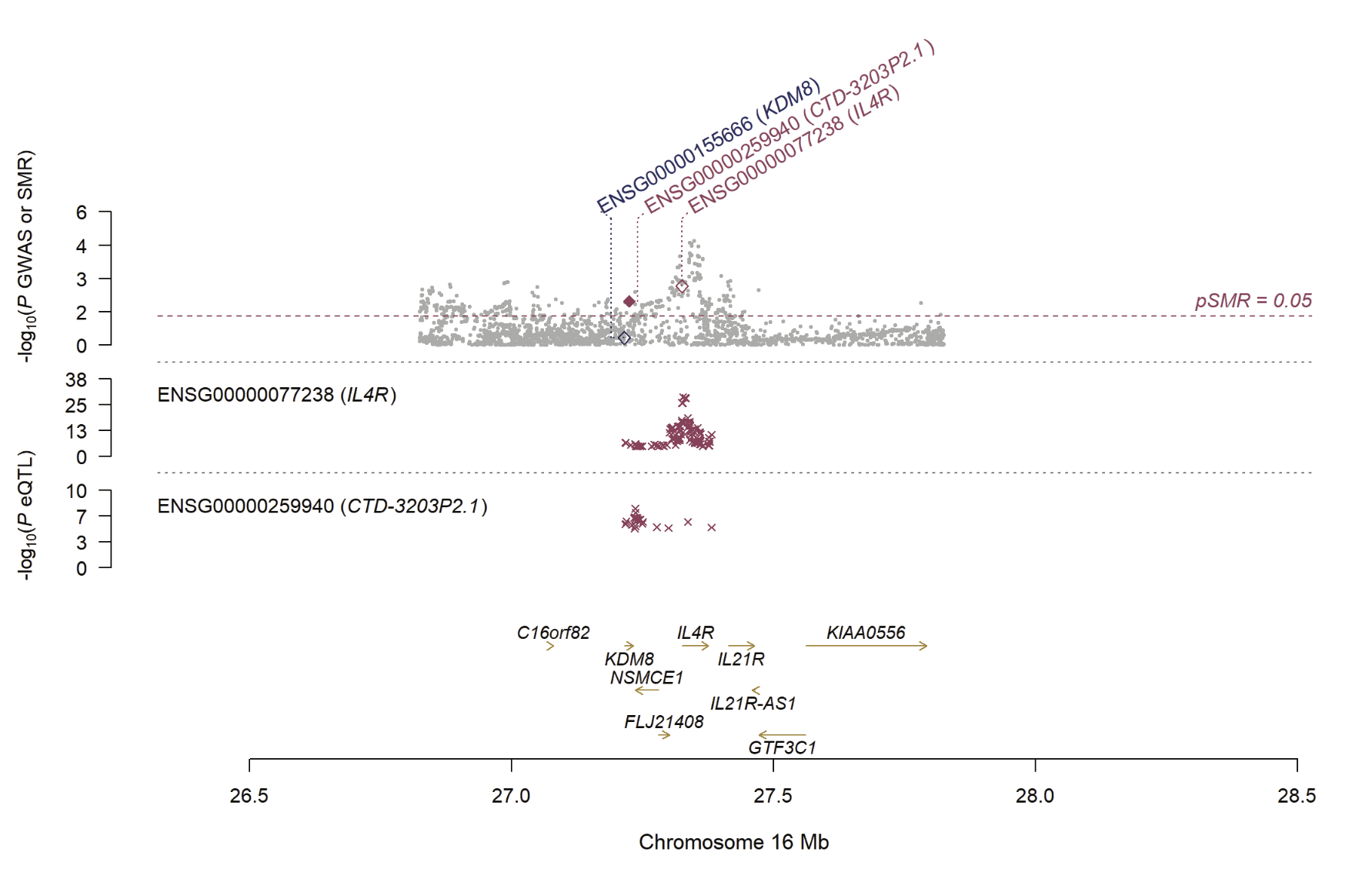


**Supplementary Figure 4.** SMR analysis identifies IL4R as a key gene linked to allergic rhinitis (AR) through gut microbiota metabolites.

SMR analysis identifies IL4R as a key gene linked to allergic rhinitis (AR) through gut microbiota metabolites. The top panel shows GWAS and SMR –log10 *p*-values with a significance threshold at *p*SMR = 0.05. Middle panels display eQTL signals for IL4R and CTD-3203P2.1. The bottom panel presents gene annotations on chromosome 16. The top SNP rs8052962 shows a positive association (b_SMR_ = 0.32, *p*SMR = 0.02), and the HEIDI test (*p*HEIDI = 0.99) supports a causal link between IL4R expression and AR.
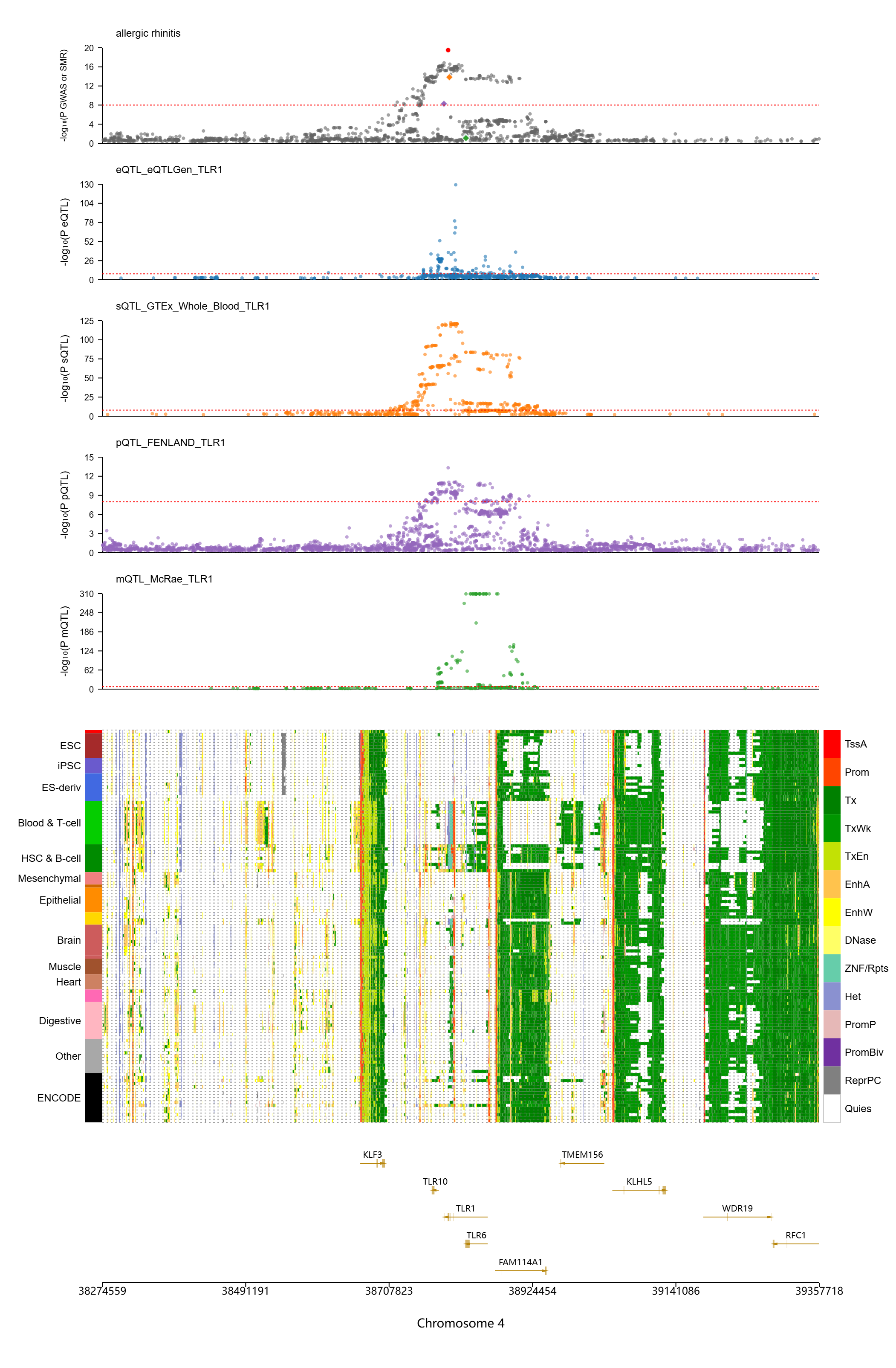


**Supplementary Figure 5.** SMR analysis indicates significant genetic associations at TLR1 with AR-related DNA methylation (mQTL), gene expression (eQTL), splicing (sQTL), and protein expression (pQTL).

The top panel illustrates the colocalization of allergic rhinitis (AR) genome-wide association study (GWAS) signals with these TLR1-related quantitative trait loci across the gene cluster. The middle panel displays tissue and cell types on the left (e.g., ESC: embryonic stem cells; iPSC: induced pluripotent stem cells), alongside a color-coded legend on the right representing chromatin states, including promoters, enhancers, transcriptionally active regions, heterochromatin, and quiescent states. The bottom panel shows gene annotations and genomic coordinates, highlighting candidate functional regions within the locus.

## ScRNA-seq Analysis Results

To validate the functional roles of the core risk genes we identified within a relevant disease tissue, we conducted an in-depth analysis of single-cell RNA-sequencing (scRNA-seq) data from the patients with AR and controls (GSE273975). Our analysis first revealed a distinct pathological state within the AR nasal mucosa. Principal Component Analysis showed a clear separation between cells from AR patients and healthy controls at the transcriptomic level, indicating a fundamental shift in the cellular ecosystem (Supplementary Figure 6A). Cell-type annotation further elucidated the nature of this alteration: compared to healthy controls, AR tissues underwent dramatic cellular remodeling, primarily characterized by a significant expansion and infiltration of Epithelial cells and Natural Killer (NK) cells (Supplementary Figure 6D-E). This shift in cellular composition, marked by the infiltration of immune cells, creates a pathological microenvironment conducive to the pro-inflammatory action of the identified risk genes.

Critically, we observed that the core risk genes were specifically activated within this remodeled cellular microenvironment. As a key receptor driving Type II immune responses, IL4R expression was significantly upregulated in AR patients. This upregulation was particularly concentrated in the expanding cell populations—NK cells and epithelial cells—providing strong evidence that elevated IL4R expression enhances the responsiveness of these key cells to upstream allergic signals, thereby amplifying the inflammatory cascade. Concurrently, IL1B, another potent pro-inflammatory cytokine, was specifically overexpressed in the B cells of AR patients, directly contributing to the local inflammatory milieu (Supplementary Figure 6F-G).


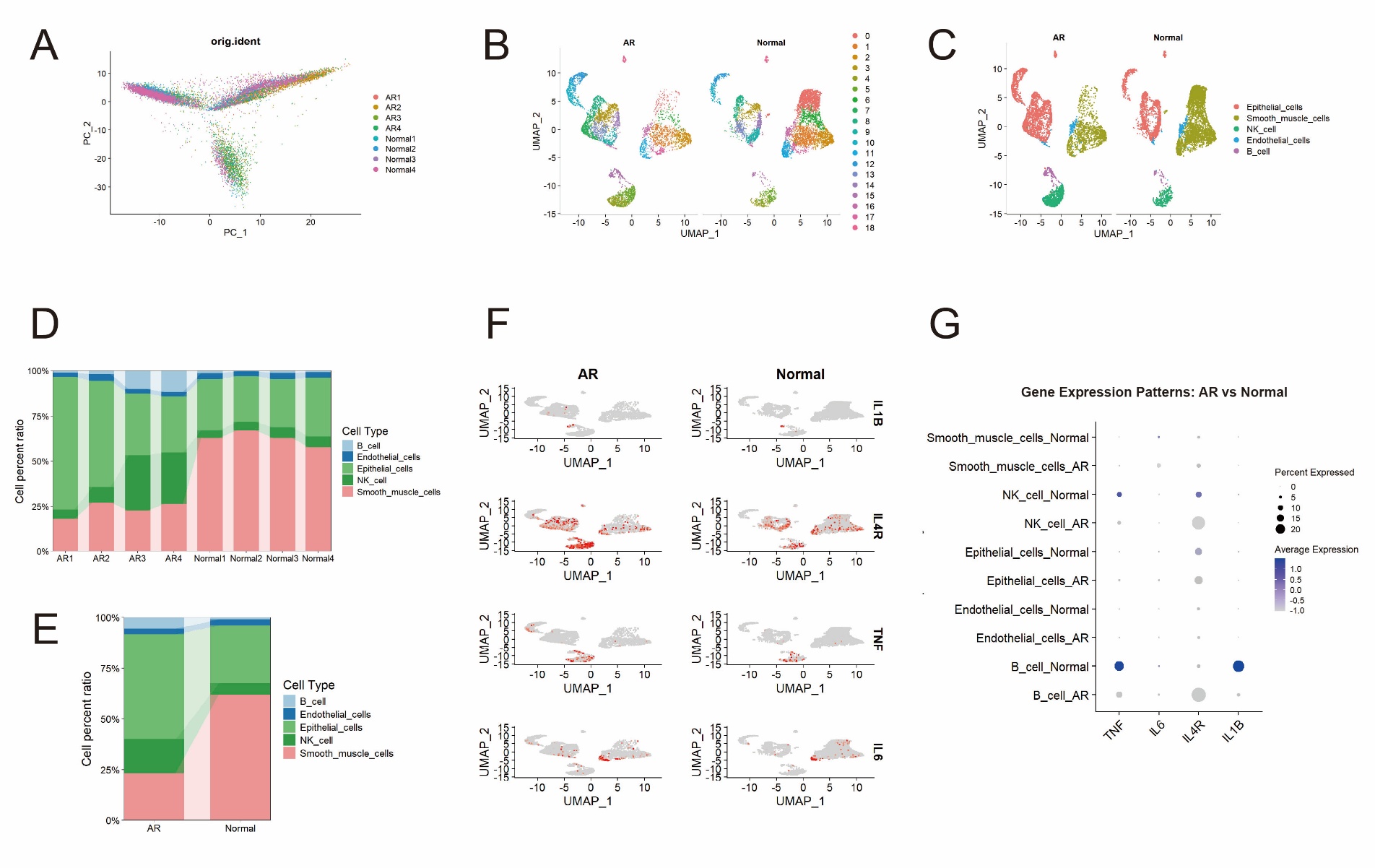


**Supplementary Figure 6. Single-cell transcriptomic profiling of patients with allergic rhinitis.**(A) Principal Component Analysis (PCA) of all cells, showing a clear separation between cells from allergic rhinitis (AR) and normal samples at the global transcriptome level; (B) UMAP dimensionality reduction plot colored by cell clusters (0-18) and sample origin; (C) UMAP plot colored by annotated cell types, with five major cell types identified; (D-E) Proportions of cell types in the AR and Normal groups, showing an increased proportion of Epithelial cells and NK cells and a decreased proportion of Smooth muscle cells in the AR group; (F) Expression distribution of the core risk geneson the UMAP plots for the AR and Normal groups; (G) Dot plot showing the expression patterns of the core risk genes across different cell types and groups.
